# Behind the Counter

**Published:** 1893-04

**Authors:** 


### BEHIND THE COUNTER.

### No. 4.

The holiday rush has subsided, and we are improving the lull in business by taking an account of our reduced stock. One would scarcely suppose that the kitchen department would be extensively patronized in the high tide of shopping preliminary to Christmas, that carnival time that fills all Christendom with rejoicings, but so it is, for the quality of gifts is as various as the fortunes of givers, and I oftentimes think that those simple and inexpensive tokens of regard which find expression in some useful utensil needed and appreciated in the home, have more heartiness in them than the tinsel nick-nacks and costly gew-gaws that constitute the bulk of interchangeable Christmas gifts, gauged not unfrequently, by no higher sentiment than expectant reciprocity, with an eye to commercial values. Shall I give you some account of my last and more recent Christmas? I've a mind to do so, for it points a moral that should not pass unheeded. Two or three examples will suffice. Only a few short months ago I was looked upon as the favorite of a rich old uncle, engaged in the profitable venture of gold mining in some far away Eldorado of the West.

In a former paper I mentioned somewhat of his temptations, ventures and disappointments, and my consequent resolve to be at least self-supporting. Well, then, to look backward a little more than a year, I then counted among my Christmas holdings a handsome photograph album, infolding a cabinet likeness of the handsome giver. Also an exquisitely inlaid escritoire; also a quantity of rose-tinted note paper and envelopes in a velvet receptacle that shut with a silver clasp, with a pearl handled gold pen included, as if I was to spend my whole life writing billets doux. These from as many young gentlemen of no mean aspirations in their several spheres of usefulness. The first giver was a clerk in a bank, whose employment for the most part was counting and denoting parcels of soiled greenbacks; the second was a rather raw disciple of Blackstone, who held his prominence in expectancy: the third was a divinity student, whose mentality was absorbed in the endeavor to reconcile the fictions of theology with the truths of science. They made frequent calls at my uncle's and professed an unusual interest in me, but when my employment indicated a disadvantageous change of circumstances, their interest in me gradually subsided, and, as I opine, their holiday outlays have been long since set down to profit and loss. As a consequence, the late Christmas passed without so much as the simplest reminder that we had ever known each other, now that the supposed heiress had given place to the struggling shop girl.

I ought in justice to mention an occasional visitor in the person of a self-sustaining young gentleman, whose father was a life-long friend of my uncle, with a like experience in similar enterprises, so that at his demise little remained to the widowed mother. Walter, the younger of the boys, was only a stripling then, but he contrived to stem the tide that thus early set in against him. By severe application, with the aid of night schools and private tutelage in the higher educational branches, he managed to fit himself for almost any position where intelligence and culture are brought into requisition. On Christmas a year ago I received under the direction of his well known hand a cheap edition of "Percy's Relics of Ancient Poetry," and gave it place on a side table among the more pretentious greetings of the season, where it served as a mark for the pleasantries of some of my self-admiring visitors. "Ah, Percy's Relics! Do you intend to cremate them, or will you consign them to Potter's Field?" inquired he of the photo-album, with mock gravity. "Poor Percy? So he's passed in his chips. Will you go into mourning, Miss Pauline?" asked the escritoire contributor with an air of solicitude. "He was ill-conditioned enough in this world, perhaps he will fare better in the next," remarked the pen and paper youth with a sardonic grin.

Without betraying the emotion which this ill-conceived raillery gave rise to, I rejoined that it was not always wise to estimate either men or books by the quality of their external covering, which brought about a sudden change of subject, as well as demeanor.

Suffice it to say that this admirable compilation of poems has been productive of more real enjoyment to me than any one thing that I can call my own.

But as to my latest Christmas, it was a very quiet one in strong contrast with its forerunner, but to me far more enjoyable. My good friend Walter bore me kindly in mind as before, and the self-supporting shop girl was not without some tokens of a friendship that survives the vicissitudes of fortune.

But let me return to my pots and kettles. We have concluded our account of stock, an easy task in comparison with other departments where cut goods are to be measured and innumerable small packages inspected, classified, told off and set down under different heads, in a dizzy wilderness of figures, involving the labor of days that stretch far into the night.

I have had something hinted to me to-day that has caused me considerable anxiety. No, I am in no immediate danger of losing my place, but it has been arranged to transfer me to the hosiery counter on the main floor, with an increase of fifty cents a week to my salary, as we girls choose to dignify our scanty allowances, but I so dislike to leave 13, who has always been so kind and considerate toward me, that I would gladly renounce any advantages of promotion to remain with her, yet I suspect I am chiefly indebted to her good words for the contemplated change, and 13 herself advises it. She says we can take our mid-day lunches together as heretofore, and the half-hour will enable us to fill it to overflowing with womanly gossip. Besides she has invited me to a tea with her at her snug little apartments, as she calls them, whose altitude affords glimpses of the ever changing Hudson and the picturesque Jersey shore in the distance. This is a pleasure I am holding in anticipation to be realized at no distant day.

